# Stories of Shame, Stories for Shame: Fiction and Self-harm

**DOI:** 10.1353/lm.2025.a975552

**Published:** 2025-01-01

**Authors:** Veronica Heney

**Affiliations:** Discovery Research Platform for Medical Humanities, Durham University

**Keywords:** Self-harm, shame, *Anesthesia*, representation, character type

## Abstract

That self-harm is a shameful practice is often taken for granted. However, recent sociological work has called attention to the way this shamefulness is actively constructed through narrative. This essay takes up that call, with a particular focus on fictional representations. It takes an interdisciplinary approach, weaving together a close reading of Tim Blake Nelson’s 2015 film *Anesthesia* with data from interviews of people with experience of self-harm. Specifically, it attends to the location of shame in a “type” of self-harming character, the significance of visibility and exposure in narratives of self-harm, the function of genre in invoking or communicating shame, and the uncertain relationship between destigmatization and the enforcement of norms. It thus explores shame as contingent, relational, and actively brought into being.

## Introduction: Shame and Self-harm

The shameful nature of self-harm is often simply taken for granted. Philosopher Nancy Nyquist Potter confidently characterizes self-harm as “particularly secretive and shameful,” more so than gambling or alcohol addiction.^1^ There is certainly ample confirmation that self-harm is considered by the general public to be shameful, and is at times experienced as shameful by those who practice it.^2^ Yet such research rarely explores the dimensions of self-harm’s shamefulness; what, precisely, is shameful about self-harm?

Amy Chandler has critiqued the overreliance on shame as an explanatory framework for self-harm, noting that it “closes down alternative explanations and experiences which might interpret the scars, wounds, and marks of the practice differently.”^3^ Kesherie Gurung similarly suggests that our ability to comprehend the full range of self-harm’s meaning and significance to those who practice it is limited by the fact that, so often, “self-harm narratives incorporate messages of shame and disgust.”^4^ Here the role of narrative comes to the fore. If we are to regard self-harm as not inherently shameful, then the ways shame becomes attached to self-harm, or manifest within experiences of self-harm, are important to attend to. In this essay, I focus on one specific type of self-harm narratives: those that occur in fictional representations in film, television, theater, and literature. I suggest that fictional representations are one way through which self-harm is established as shameful, thus creating a possibility for it to be experienced as such.

I draw on Sara Ahmed’s description of shame as “an intense and painful sensation that is bound up with how the self feels about itself … a feeling of negation which is taken on by the subject as a sign of its own failure.”^5^ Ahmed presents shame as a series of contradictions: as involving both exposure and an attempt to hide; as functioning to “turn the self against and towards the self”; as simultaneously requiring self-recognition and recognition by others; and as prompting a movement away from those others as well as an interest or investment in them.^6^ In taking up Ahmed’s theorization, I attempt to extend her assessment of “the inter-corporeality and sociality of shame experiences.”^7^ I will demonstrate that fictional representations are one of the modes or methods through which shame is socially structured and transmitted, and explore the ways shame functions not only to mark individuals, but also to associate or establish relationality. Sally Munt has described shame as a “sticky” emotion to which other emotions might be easily attached.^8^ I suggest that shame’s stickiness also functions to bring together, associate, or attach subjects (both fictional and real) with and to one another. Thus, I does not theorize shame; rather, I theorizes the ways shame might move through, or be invoked and manifested by, fictional representations in relation to self-harm.

I deploy an interdisciplinary approach, bringing together literary and sociological methods to interweave a close reading of a fictional text with analysis of qualitative data. The data in question comprises 17 semi-structured interviews with people with experience of self-harm, conducted as part of my doctoral research into fictional representations of self-harm; participants were asked about the fiction in which they had seen self-harm represented, and it was clarified that fiction referred broadly to literature, film, television, and theater. To capture the breadth and multiplicity that stretches across the different genres, I use of the term *fictional representations*. Participants discussed examples across this breadth of genres, and also reflected on these representations more generally.

Fourteen participants were white, white British, white Scottish, white Irish, white Jewish, or white European; one participant was mixed race, one was Eurasian, and one was British Asian. Nine participants were in their mid-teens to early 20s, two participants were in their late 20s, five were in their 30s, and one was in her 60s. Eleven participants described themselves as queer (5), bisexual (3), pansexual (1), lesbian (1) or asexual (1), while four participants described themselves as straight or heterosexual. Participants were given the choice to be pseudonymized or to be referred to using their own name. All interviews were audio recorded and subsequently transcribed; all participants were paid for their time, were sent a copy of their transcript, and were contacted for feedback on the analysis, following best practices in survivor research.^9^

The data analysis was guided by Natasha Mauthner and Andrea Doucet’s extension of Lyn Mikel Brown and Carol Gilligan’s Listening Method, a process which involves reflexive, sequential readings.^10^ Having identified initial key themes, I brought the interview data into relation with fictional representations. In this subjective process I used my own judgment to assess thematic areas where fiction and data together created interesting, significant avenues for further analysis and writing, of which shame was one. During the interviews, participants were not explicitly asked about shame; rather, through data analysis, I identified that shame was mentioned repeatedly, and often in ways that suggested its importance as an aspect of self-harm’s fictional representation and of the experience of reading or watching such texts. I have included in the analysis below instances where the word “embarrassment” was used, recognizing Luna Dolezal and Barry Lyons’s description of shame experiences as “varied and multiple,” and thus including a wide range of emotions, including experiences such as embarrassment and humiliation, the former often being theorized as a milder form of shame.^11^ While I did not wish to elide a valuable distinction between the two emotions, it seemed, in this case, generative to group the data together, recognizing the difficulty of enforcing firm delineations between how different individuals might describe their feelings.

Participants in the study clearly did not regard self-harm as universally or inherently shameful. Sally, a queer, white British woman in her late teens to early 20s,^12^ commented “I’ve never really felt ashamed or that guilty of self-harm…. [I]t doesn’t have the same shame as something like an uncontrolled binge would have.” Cat, who was genderqueer, asexual, Eurasian, disabled, and in their late teens to early 20s, felt that the stigma of self-harm was not inherent but rather related to place and culture. They wrote that “Self-harm still seems to be a taboo in Singapore and it feels more stigmatized than it does in the UK,” a fact that influenced decisions about whether to wear short-sleeved tops, for instance. Siobhan, a straight, white Irish woman in her 30s with a mental health disability, talked about self-harm in fiction and said: “When I was with my boyfriend and if there was ever self-harm in a film or something that we were watching[,] It was always really shameful…. [But] It’s kind of like if it’s me on my own, it just loses my interest. It’s just like, whatever.” In these quotations we can see that shame’s presence or absence might be dictated by culture, geography, relationality, or simply personal experience—when other practices are experienced as shameful, self-harm’s shame seems to disappear by comparison.

In the interviews, no single fictional representation was mentioned more frequently in relation to shame. As a result, I selected a visual text which, to me (as a person with experience of self-harm), had conveyed and invoked shame when I watched it as part of my preparation for conducting the interviews. I will discuss Tim Blake Nelson’s 2015 little-known *Anesthesia*, a film with an almost nonexistent viewership, poor public reviews, and no critical academic reception. In exploring this text alongside the interview data, I don’t seek to make a claim for its quality. Rather, it is a helpful example because it contains tropes typical in fictional representations of self-harm. In what follows, I present my own reading of the film, including the ways it demonstrates the representational tendencies discussed by participants. The film is a multi-storyline drama, tracing interconnections between a wide cast of characters. After opening scenes of a mugging victim and a stranger whose house he flees into, the film backtracks to explore how the lives of the two men arrived at this intersecting point, while also tracing the stories of a family coming to terms with a cancer diagnosis, a lawyer trying to help his childhood friend who is struggling with drug addiction, and an unhappy housewife who has found solace in alcohol. Interweaving these many narrative arcs, the film returns to the mugging, which we now understand differently. One storyline follows Sophie, a promising graduate student, who is self-harming and is helped by her professor, Walter Zarrow, who the audience recognizes as the victim of the mugging at the film’s opening.

I structure my argument based on the themes present within the interview data; in each section, having explored the views of participants in the study, I turn to *Anesthesia* to demonstrate the ways their judgments of fictional representations of self-harm in general can be seen in the specific. I also use my own analysis of the film to draw attention to the representational techniques through which self-harm is made shameful in each example. The essay thus makes an argument that is both general and specific; while it does not and cannot address the whole of fiction, the comments of the participants strongly suggest that trends present in *Anesthesia* might be present elsewhere, and the analysis of this essay might be useful for exploring other fictional representations of self-harm. The four sections of this essay argue that self-harm’s shamefulness is neither universal nor inherent to the practice itself; rather I will demonstrate that it is actively constructed, in part through its representation in fiction. As I show, participants did not simply feel the already-existing shame of self-harm, prompted by its representation, nor did they straightforwardly experience an echo of the shame felt by self-harming characters. Rather, their own shame was constructed, invited, or induced by how the texts positioned and depicted self-harm, and through the ways they, as self-harming viewers and readers, felt they were brought into relation with self-harming characters.

First, I will explore how self-harm is associated with characteristics or traits which are themselves understood as shameful, through its fictional association with a character “type.” Second, I will consider how self-harm is understood to invoke judgment, by its depiction through narrative structures predicated upon logics of exposure. Third, I will explore how self-harming subjects come to be framed within certain genres, such as melodrama, which are independently experienced as shameful. In conclusion, I will suggest that efforts to decouple shame and self-harm might inadvertently reinforce the relationship through repetition and normativity. In each case, fiction’s position as that which can mediate between the social and the individual, that which can associate and draw into relation, makes it particularly significant in establishing self-harm’s supposedly fundamental shame.

### Transmitting Shame through Character “Types”

Having already established that some participants did not regard or experience self-harm as inherently shameful, I will now explore how self-harm’s shamefulness might be established through its association with particular characteristics of personality, giving an example of how this occurs in *Anesthesia*.

One participant named Faye, a queer, white European woman in her late teens or early 20s, described “a bunch” of novels that were “supposed” to “depict teen life” in which she felt self-harm was portrayed as “really strange, foreign, and a little bit gross” and “there wasn’t a really strong connection as to this being an understandable practice. It was more like a really weird thing that broken people do.” She said that this sort of portrayal “made me feel a lot of shame” and that because “that’s the way it was seen I felt so ashamed…. they’d know that I’d have done that.” Riley, a queer white British gender-questioning woman in her late 20s, talked about different forms of shame that narratives of self-harm could engender, from the “shame because you don’t see it” to the “shame because everyone’s a nutter who does, or annoying.” Here, again, particular aspects of self-harm’s representation bring shame to the fore: Riley had previously talked about the tendency for characters who self-harmed to be annoying, describing Jenny in Showtime’s *The L Word* (2004–2009) as being “painted as like mopey and like a bit weird” and being “untrustworthy” or “manipulative.” Marie, a pansexual, white European woman in her late teens to early 20s, talked about divergent trends in cultural approaches to self-harm. In Catherine Hardwicke’s 2003 film *Thirteen*, because the representation of self-harm was “dirtier and grimier” than other tonally lighter depictions, she got the sense that “this is something like you should be ashamed of. That only like, the low of the low kind of do this, right.” She reflected that although the two divergent styles of representation touched on similar topics, the nature of their representation “steered” or changed “how you felt about them.”

These reflections further demonstrate that self-harm’s shame is not inherent. They also suggest the particular ways self-harm’s shame might be communicated or enforced by fictional representations. There is a striking convergence in the qualities these participants identified as shameful: the repeated language of brokenness, of weirdness, of being manipulative or dirty or gross. This language also appeared at moments in interviews with other participants, suggesting that its invocation might hint at the presence of shame, even when the emotion wasn’t explicitly identified. Amber, a bisexual, white woman in her late teens or early 20s who listed her mental health conditions as anxiety, depression, and borderline personality disorder, talked about a book that portrayed self-harm well because “it just makes you really empathize more with that character. Rather than it being oh this character’s doing a really weird, gross, awful, selfish thing.” In contrast, when watching documentaries about self-harm, she felt “discomfort” when people talked about it “in an indulgent way” or in a way that seemed “immature.” Margaret, a heterosexual white, Jewish woman in her 60s from a working-class background who had lived with chronic clinical depression, talked about reading Angela Carter’s 1971 novel *Love* and feeling “quite a lot of guilt and quite a lot of shame.” She felt the novel encouraged an awareness of the way those around her had likely seen her “as someone behaving in a, an extremely weird way.” Jon, a heterosexual white man in his 30s, talked about wanting to distance himself from “the *ah woe is me* narrative” or language that seemed “histrionic.”

These varied comments present an intriguingly consistent picture of the type of self-harm or self-harming subject which is experienced or understood by participants as particularly shameful. Nonnormativity certainly comes through strongly, but I am more interested in the particular tenor of this “weirdness,” which seems to be associated with the “failure” of being broken, the abjection of being dirty, the moral collapse of being manipulative or indulgent, and the callow solipsism of being immature or histrionic. This shame is not a universal—or rather, it is not a shame which functions universally; this shame is specific. It is enacted through characteristics which are not necessarily inherent to self-harm but are associated so closely and repeatedly with it that they come to *seem* inherent. This association is in part accomplished through narrative: as the participants make clear, narrative functions to establish a particular version of self-harm, one that is frequently laden with shame.

Turning to *Anesthesia* allows consideration of *how* characters come to take on shameful characteristics in ways that enforce or enable their association with self-harm. The self-harming graduate student, Sophie, is introduced to the audience sitting alone in the university cafeteria.^13^ The orchestral soundtrack fades away as another student walks past and casually snags the seat opposite her, to which Sophie responds angrily, challenging him on why he failed to ask permission to remove it. When he asserts that it was obvious she didn’t need the chair she responds, “Who are you to determine what I need?” Her point seems fair, but also unnecessarily self-righteous: it is, after all, just a chair. The formality of the verb “determine” is out of place for a student cafeteria to the point of being pretentious. The interaction escalates, and in every back-and-forth Sophie seems more absurd, more unsociable, more self-indulgent in her refusal to let go of a minor inconvenience. Although the other student is a little rude, Sophie’s response seems disproportionate, such that when he responds angrily it’s hard to entirely sympathize with her, as she refuses to accept his apology or to simply let the interaction go.

The scene compellingly matches the dimensions of shame identified by participants: weirdness, brokenness, immaturity, over-reaction, manipulation. That Sophie self-harms will not be revealed for another 20 minutes, and yet the traits associated with such behavior are already well-established; when watching I found myself experiencing vicarious embarrassment simply watching her outburst, before I had any sense that the character self-harmed. This temporal structure within the narrative constructs the association which participants identified: the already-established shame not only communicates the shame of self-harming, but establishes it as inherent to the person who self-harms, part of a broader failure of personality rather than contained within a single act. Sophie is already shameful; her self-harm, when finally revealed, simply falls into place within this explanatory frame.

This is the creation of a type—a self-harming character becomes shameful not simply because they engage in a practice or act of self-harm, but because they are the type of character (or person) who would self-harm. As an example, consider a comment by the participant Jon: noting that he could not remember if a particular play depicted self-harm, he said that the “kind of person in a Simon Stephens play *would* self-harm” (italics added). The interview data demonstrates this association between self-harm and shameful characteristics is not simply information which a text may or may not communicate; rather, it is a force which acts to create relationality. In establishing a self-harming “type,” fictional representations draw readers and viewers who self-harm into relation with those characters.

Siobhan, talking about the shame she felt watching depictions of self-harm alongside other people said, “It makes me embarrassed, kind of. Because it’s like maybe I’m doing similar things for similar reasons.” Blanche, a lesbian, white Scottish woman in her late teens to early 20s, recalled the first season of FX Networks TV show *American Horror Story* (2011–), in which the Harmon family’s eldest daughter Violet is established early on as drawn to darkness when she becomes more positive about their rental house after discovering it was the site of a murder-suicide. Blanche said, “I was like, oh my god if people see this. And they think that this is like, this is only what self-harm is. I was like, Oh man, this is, this is really embarrassing. It was like I don’t want anyone to associate me with this, like, bullshit … this like, edgy, indie teen bullshit. I was like, Oh, this is embarrassing.”

These comments demonstrate how shameful depictions of self-harm are not mere descriptions, containers of information: rather they are vectors that connect and transmit shame. Kaye Mitchell discusses “the troubling intersubjectivity and transmissibility of shame,” which Barry Sheils and Julie Walsh suggest “potentially rips through the contours of the subject, throwing into disarray the formal distinctions between inside and outside.”^14^ In participants’ comments the boundaries of the self-harming subject become blurry, as reader-viewer and fictional character become inextricably entangled. Yet there is also a demarcation. If we consider the comments outlined earlier, wherein participants felt that fictional representations presented self-harming subjects as “weird” and nonnormative, we can see that this functions to delineate these subjects as different to, and separate from, others around them. Thus, while shame functions in one direction to associate, it simultaneously works to mark it out as extraordinary. Through becoming associated with a particular self-harming “type,” individuals are singled out as different, as weird, as broken, as solipsistic and manipulative and immature: their existence as individuals, even as subjects, is suspended and they become one among many, an example of a category, and specifically a shame-laden category.

This, too, is explicated through Sophie’s character in *Anesthesia*, as we see how viewers might be drawn into relation to her, without gaining a sense of her subjectivity. It is clear, as we are introduced to her, that she is the character upon whom this storyline centers: the camera pans across the faces of other students in the cafeteria laughing and talking before coming to focus upon her face as she sits alone.^15^ She is amongst her peers but not part of them. As the uncomfortable incident over the chair plays out, students in the background of the shot seem increasingly to be aware of what is happening. Yet when the young man shouts at her, no one intervenes, no one checks on her, no one helps her to pick up the chair which he has knocked over. The students around pause and become quiet as the encounter unfolds, but conversation resumes the moment it concludes.

In the next scene, after Sophie has packed up her books and left the cafeteria, she is sitting in a bathroom stall alone. She has been marked by the film as isolated from other characters—and also from viewers. We can hear her breathing, but there’s no opportunity to know what she’s thinking, or how she feels. Rather than facing her head-on, the camera angle is voyeuristic, peering down so that we see the top of her head more than her face. There’s barely time to wonder what she’s doing before the camera cuts to a different storyline. We have been looking at Sophie, but not in such a way that we might really see her or at least see her subjectivity. Instead, the cinematography suggests she has little subjectivity to see. She has become object-like, an example of a type.

It is easy to imagine for viewers who self-harm that being associated with Sophie is insulting, frustrating, even inaccurate. The film creates a social type, a structure of knowledge into which self-harm, and all self-harming subjects, might be placed. While my interview participants referred to a wide variety of other texts, *Anesthesia* offers one example of this phenomenon, and demonstrates how it might be accomplished. To participants, it mattered less that their experiences weren’t *actually* similar to these characters; they knew that they risked being read, by others, as belonging in the same category. They came to feel shame simply because of perceived similarity and association, rather than their own feelings of identification. Through narrative’s ability to establish social relationality, the participants’ own ability to exist or be recognized as full human subjects was diminished, and they came to share in characters’ positioning as object-like examples of a shameful type.

### Visibility, Exposure, and Judgment

I now turn to participants’ comments on visibility and exposure as components of the way narratives of self-harm constructed self-harm as shameful and prompted feelings of shame in those reading or watching them. In particular, I note how fictional representations position self-harm as something which invites judgment through their narrative structure and, using *Anesthesia* as an example, discuss how moments of “discovery” are invested with shame by their association with embodiment, vulnerability, immaturity, and oversharing.

The significance of visibility can be seen in Margaret’s account of reading *Love* (1971), as she talked about a self-harming character who was “seen totally from the outside” throughout the novel. She commented that because of this framing, and the fact that the character was in her early 20s, “I did kind of equate it with how people might have seen me at that age as well. And I felt quite lot of guilt and quite a lot of shame.” She echoed this phrasing later, talking about how she thought that the text invoked “how people around me perhaps felt looking at me.” Visibility is frequently foregrounded in theoretical literature on shame. Mitchell suggests that “shame itself is so much a matter of visibility and concealment,” while Dolezal describes the affective experience of shame as involving “an intensification of the body’s surface and its visibility.”^16^ In addition to visibility, shame is often specifically associated with exposure. Ruth Leys even suggests that shame is *identical* to exposure: being shamed is the experience of being revealed as in some way failed or flawed.^17^ It is not the failure itself, which is shameful, it is the uncovering of that failure by or in front of others, whether real or imagined.

Lou, a bisexual, white British woman in her 30s, talked about the film *Thirteen*, which included “a narrative around being discovered.” She suggested that there was a tendency for fiction to depict a character self-harming, and then to depict an intervention that brings about the cessation of that behavior. She said that these sorts of structures didn’t encourage her to ever talk to people about her self-harm “because [in] the stories that I see it’s a secret and it’s something shameful.” Rather than being invested with shame through association with other shameful traits, here self-harm becomes shameful through the narrative structure within which it is placed. Self-harm is shameful because it is discovered, because what was secret has been exposed and is seen as unfit. This could be somewhat circular; it could equally be argued that self-harm must be discovered *because* it is shameful, which is also why it was hidden. My point is not to suggest a sequential logic but to emphasize the mutually reinforcing nature of such a construction: the more self-harm is associated with secrecy, the more it is inevitably understood as shameful, no matter what came first.

In *Anesthesia*, the moment of discovery is self-prompted, but it is no less a moment of unveiling. In the midst of a somewhat abstract conversation about philosophy with Professor Zarrow, who is her advisor, Sophie stands up and, without saying anything, pulls up her sleeve to show the scars on her arm, and then pulls down her jeans to reveal the scars on her thighs.^18^ A sense of exposure pervades the scene, in part because of Professor Zarrow’s response, as he asks, “Why have you done this to yourself, Sophie?” Even when said kindly, this question implies judgment: Sophie has done something unacceptable and must explain herself. For all that she *chose* to share her self-harm, she has still been exposed.

This is made even more explicit by the doubling of exposure here: to show her scars, Sophie must undress; to expose the scars is to expose flesh, to come dangerously close to nakedness. The silence as she does so, and the jerky awkwardness of her movements, emphasizes the inappropriateness of undressing in her advisor’s office. To me, while watching, shame infused the scene and was immediately present in my own embodied response. The choice to undress—a choice that belongs both to Sophie and to the film itself—enacts the way that self-harm is inherently exposing, because making its presence known always involves a literal or metaphorical becoming naked. In part, this reading of the film simply reinforces interviewee Lou’s point that exposure is one way self-harm is established as shameful. However, it also extends this analysis by demonstrating the precise dynamics of self-harm’s discovery through which it is associated with inappropriate, uncomfortable vulnerability and both literal and emotional nakedness, and thus social failure or unacceptability.

Rosa, a white woman with a disability in her late teens to early 20s, described a novel where self-harm is “always shown … in scenes where they are naked,” which suggested that the practice either “links to sex or links to being vulnerable.” She went on to talk about her sense that if a film or book was in some way connected to self-harm, “it’ll have a sex scene in it. Or a link to saying about sex.” Although there are some exceptions to this observation, I found it insightful. She thought the connection between self-harm and sex might derive from the fact that sex, especially outside of marriage, has often been “condemned in the same way that people still think self-harm is wrong, morally wrong.” While her invocation of morality echoes the sense of judgment I’ve described above, her comments also suggest the presence of shame. The shame of self-harm is in some way the shame of sex, of embodiment out-of-place, of bringing often silent, private, bodily experiences and considerations into public view. To confess self-harm is to confess a fact of embodiment; it is to bring matters of the body to the fore, to reveal a truth about your body, to make it visible whether literally or figuratively. It is perhaps unsurprising that within such a structure, shame seems impossible to avoid.

To share her secret Sophie must make herself vulnerable twice over and thus is shamed twice over: by the act of self-harm itself and by the way she chooses to make it visible, exposing more than necessary. Once the scars on her arm are apparent, there is no narrative or practical need to show those on her thighs, and yet she chooses to pull down her trousers. Over and over the scene lingers in excess, in superfluity. This echoes the ways participants felt popular narratives portrayed the nature of self-harm: as a melodramatic, excessive response, a failure of emotional control that signals shameful immaturity. This dynamic is reflected in the construction of the scene, which isn’t one of drama or high tension; there’s no accompanying soundtrack to build mood or create shock value. Yet in some ways, this absence of sound makes the sense of exposure, and accompanying shame, even greater. Sophie’s revelation is out of place, out of step: it is not just what Sophie exposes that is shameful, but the act of exposure itself: a sudden jolt of intimacy where previously there was academic, philosophical discussion of “precepts.”

The jarring shift from intellect to embodiment is emphasized through the scene’s construction. As Sophie pulls back her clothes, the camera moves to show close-up shots of the scars, as if to double the exposure. This is intercut with an initial wider shot of her whole body as she prepares to undress, locating her firmly in the office setting, and then later with a shot that pans up to show her hunched over, hair covering her face but her thighs bare. These shots of Sophie are interspersed with shots of Professor Zarrow, who remains unmoving. He is shown in two different aspects, but both capture him sitting firmly behind his desk, retaining his professional mode. He looks at her; she is looked at. She is shamed, he and we sit in judgment, we are embarrassed on her behalf. Through positioning self-harm as that which traffics in exposure, that which is reinforced is shameful, and moreover that which is practiced by such people is embarrassing: they are exhibitionists, who exist and position themselves as objects to be looked at and judged.

Narrative structure here aligns with the specifics of the scene, its tone, shot construction, and physicality, to create a pattern of association whereby self-harm is positioned as inevitably and inherently shameful by its function as that-which-is-confessed and by its association with embodiment, vulnerability, immaturity, and excess. While *Anesthesia* is a visual text, and thus visual evidence might be assumed to take on particular significance, both Margaret and Rosa were describing a similar effect from written texts, suggesting that visual evidence (whether shown or described) comes to the fore across fictional forms. Moreover, what is both judged and found shameful is not simply self-harm, but the person who self-harms. It is no surprise that this way of representing self-harm—via the mode of exposure—might suggest to self-harming subjects who read or view such texts that they should understand themselves as shameful.

### Genre, Artistic Failure, and the Threat of Imitation

I now turn to participants’ suggestion that certain representations of self-harm invited shame not simply through their depiction of self-harm, but also through their broader artistic failure, or their failure to capture the meaning of experiences of self-harm. Drawing on *Anesthesia* to demonstrate the ways self-harming characters come to take on the characteristics and artistic failures of certain supposedly shameful genres, specifically melodrama, I will explore the possibility for shame to act as a *connecting force* in relation to accusations of imitation.

Sometimes participants talked about finding representations of self-harm to be of poor quality and therefore shameful or embarrassing. In referencing *American Horror Story*, Blanche said that she felt “second-hand embarrassment” from the thought that “a writer has actually sat down and wrote this scene and thought that this was going to be, this was going to have like shock value.” Blanche’s comment conveys derision, a strong sense of the mortification when watching a text that, to her, self-evidently failed in what she perceived to be its ambitions. A similar sense comes across in Lou’s discussion of James Mangold’s *Girl Interrupted* (2000), a film that represented an experience close to her own. She said that although she was embarrassed because the film was “cliché,” she also felt that the film was in some way “precious”; she held on to the “poetry” and the “romance and the sentiment” of the film, feeling connected to it. If a film itself is judged to be in some way shameful by its viewers, then to be attached to it, to be interested in it, is particularly shameful. This is not to say that all viewers of *Girl Interrupted* judged it to be cliché, but it is clear Lou felt this was a possible or likely reception of the film. Her case demonstrates the potential for shame to multiply: the shame of being invested in a “bad” text echoes the shame of being invested in the “bad” practice of self-harm.^19^ Moreover, for both Blanche and Lou the perceived failures of the fictional representations are felt so strongly that the shame seems easily to attach itself to them rather than remaining with the text’s author or creator.

Talking about the experience of watching self-harm in films, Siobhan said, “It makes me embarrassed, kind of. Because it’s like maybe I’m doing similar things for similar reasons…. I think maybe misguidedly, I’ve kind of added like a mysterious factor to it when there’s nothing really mysterious…. I think I’ve romanticized it a bit maybe.” Siobhan’s comment highlights once more the way the shameful characteristics of self-harm might spread beyond the bounds of fiction and attach to self-harming subjects in reality, echoing my earlier analysis of shame’s ability to connect, to function as a vector. Yet if we return to Blanche, we can see an intensification of shame’s transmissibility, this time in *self-harm*’s transmissibility. In talking about when she was younger, before *American Horror Story* had aired, she said: “I sometimes maybe felt like a little bit better about the fact that it didn’t, that it wasn’t depicted elsewhere, because I didn’t, I almost didn’t want people to think that I was doing this because oh I’m literally just copying something I saw on TV.” This is not an uncommon accusation: that those who self-harm are simply copying something fashionable, that they are not genuinely distressed but simply melodramatic and attention-seeking. Abigail Bray has noted the popular tendency to theorize disordered eating “as the direct result of the consumption of media representations of idealized thin femininity” made possible by the (usually female) anorexic subject’s inability to maintain objectivity or distance while reading.^20^ Blanche’s comments echo this framing, wherein a reader who “copies” a fictional act of self-harm shows themself to be overly susceptible to the false, fictionalized “appeals” of self-harm. Within such a paradigm, shame is both invoked and transferred: to consume a representation of self-harm is inevitably to invoke the shame of being interested not only in self-harm but specifically in its depiction. Thus the threat of what participants judge to be an aesthetically bad or “failed” depiction intensifies; it implies the self-harming subject is not only easily influenced, but is particularly influenced by texts they perceive to be “bad,” and imagine that others might interpret similarly. They imagine that others will judge them as caught up in the superficial intensity of silly melodrama, rather than either engaging with legitimate art or experiencing genuine distress.

Turning to *Anesthesia* demonstrates how a fictional representation of a self-harming character can be narratively positioned to take on what are perceived to be the shameful qualities of the text’s broader genre, replicating within the film the relationship which self-harming subjects beyond it are imagined to engage in. In this case, the shame of a culturally denigrated and supposedly aesthetically “lesser” genre becomes the shame of self-harming subjects, through the narrative structures whereby self-harm is positioned and understood.

*Anesthesia* is a sentimental melodrama, a genre with a long history of being judged as embarrassing and shameful, albeit in complex and contested ways.^21^ Its reductive, universalizing message about human connection and the intertwined nature of our lives aligns it with common (if exaggerated) characterizations of melodrama as “naïvely sentimental [and] morally simplistic.”^22^ Sophie’s storyline contributes to this by shifting the character from a position of alienation and suspicion to one of relation and trust. This movement is seen clearly in the scene with her therapist which follows her revelation, a few minutes further into the film’s labyrinthine passage of multiple storylines.^23^ When asked to explain her self-harm, Sophie responds:

The world has just become so inhuman. Everyone’s plugged in, blindingly inarticulate, obsessed with their careers. I can’t talk to them. I fight them. I want to destroy them. I crave interaction. I crave it. But you just can’t anymore. They pull their devices out for every little thing, to reinforce their petty, convenient notions, to decide where they’re gonna shop, what they’re gonna eat, what movies they’re gonna watch, everything they ingest…. I’m just as bad as they are…. I’m, I am so fucking lonely. Why is the world so base? Why is it so insensitive? Why is it so selfish? Why am I? I am not for this world.

This speech is the occasion in which the film allows Sophie to most clearly explain what self-harm means to her. Yet the monologue veers away from self-harm, instead making broader, more general social commentary.

The contradiction of this moment is that Sophie is making a critique which the film essentially supports. Indeed, the film positions Professor Zarrow as its hero in part by making his weekly tradition of bringing his wife flowers central to the plot, and thus preaching the value of small kindnesses and human connection. Yet while the film aligns with Sophie’s broader critique about the dangers of dis-connection, it positions *her* as mistaken. Rather than speak of personal difficulties with friends or family, Sophie makes a critique about what she sees as fundamental human nature: that the world is “base” and “insensitive.” She treats her peers with derision, and while she says, “I’m just as bad as they are,” the structure of the monologue belies her words as she repeatedly uses her ability to critique the society around her to set herself apart from it. There’s a grandiosity to the speech, in its language, its tone, the scale of its critique, through which Sophie becomes pompous rather than insightful. She positions herself as wracked with self-loathing, but comes across as self-satisfied. The speech echoes the excess which characterized her previous disclosure of self-harm; in both cases, Sophie seems too much.

Mitchell notes that the act of confession itself is shameful “if read as ‘oversharing’ or narcissistic,” as there is an inherent shamefulness to “the staging of vulnerability,” particularly if it might involve excessive emotionality.^24^ From this we can consider that there is a double shame at work in Sophie’s storyline: first, the shame of self-harm itself or of being alienated, lonely, and socially inept and thus turning to self-harm. Then there is a second shame, the shame of shamelessness, as Sophie exposes herself beyond what’s necessary, and critiques her peers’ selfishness without recognizing her own solipsism. Within this doubling the first shame becomes not just magnified but justified, deserved, because of the second shame’s presence.

Even as the structure of the film seems to encourage a sympathy with Sophie’s concerns, it simultaneously emphasizes their insignificance through her positioning relative to other characters. This is particularly the case with regards to Professor Zarrow, whose death is the culmination of the narrative and functions to draw the many strands together. Viewers know he will be gravely injured from the opening scene, as his stabbing frames the film, and thus the dynamic of Sophie’s storyline is thrown into sharp relief. It may seem that Sophie is the one in danger, who is in pain, yet if we consider that imminently the professor will be attacked and near death, her concerns seem trivial and their rhetorical grandiosity becomes even more absurd. Once again, self-harm itself is not simply straightforwardly shameful, but is also associated with a shameful way of being in and responding to the world.

Ironically the film itself is hardly sophisticated, often reaching for significance and falling into sentimentality. Yet even within such trite fare, Sophie is positioned as the character who stands in for the film’s own failings: its inward-facing, tritely emotional dramatization. Sophie is associated with the supposed failings of sentimental melodrama which *Anesthesia* exemplifies (for all the broader genre is frequently more complex, nuanced, and meaningful) through her presence in the film, and then again through her own actions and rhetoric. This invites the assumption that self-harming subjects beyond the bounds of the fictional representation might be similarly caught up, entranced into imitation through the attractions of melodrama. To be associated with Sophie, for instance through a shared experience of self-harm, is to be living within this generic frame of naïve, sentimental melodrama, to be so caught up in its pathos that you foolishly imitate the behavior it depicts, whilst those around you are granted significance and insight. *Anesthesia*’s depiction of Sophie as easily influenced, immature, and insignificant gives weight to participants’ critique of failed representations and their concern that artistically poor, melodramatic texts reflected poorly on those who were assumed to imitate them.^25^

### Normativity, Repetition, and Concluding Thoughts

In concluding, I will note one final complexity in the relationship between shame and self-harm: the possibility that attempts to remove shame might instead simply shift or even proliferate it. While Dolezal argues that the relationship between shame and stigma remains undertheorized, for my purposes here I note that social structures through which certain acts become “stigmatized” might also initiate feelings of shame.^26^ Writers, public figures, and researchers frequently frame narratives of self-harm as acceptable only if they end in recovery, and indeed even suggest that discourses of hope and recovery might hold the potential to reduce the stigma of self-harm.^27^ Yet there has been much criticism of general trends of mental health “storytelling” and of the valorization specifically of recovery narratives. Anne O’Donnell, Lydia Sapouna, and Liz Brosnan have explored how certain stories or testimonies are invited into professional spaces (usually “narratives of recovery” which feature redemption for healthcare workers), while certain other stories are excluded: those told by survivors who are deemed to be “too ‘mad’ or too angry.”^28^ Such concerns alert us to the possibility that seeking to tell narratives which reject self-harm’s shame or shamefulness might result in sanitized portrayals which simply restigmatize the forms of self-harm which fall outside of these narrow bounds of acceptability. This is particularly significant given the way that acceptability and its exclusions so frequently act unevenly across structures of race, gender, and class.

The risk of positioning recovery as a norm was reflected in Lou’s experience, who noted “I can’t think of a film actually where there’s a character who uses self-harm who then by the end of the film, normally they have stopped…. But actually, that’s not particularly helpful because it’s, it’s fantasy. And the flip side of that is that sense of potential shame and kind of confusion around not fitting into what the story is telling me.” Lou’s point is not that some narratives end with recovery, but that they all do. There is a sense of the significance of repetition: over time, over successive films or TV shows or books, Lou has engaged with many narratives of self-harm and has found only one form of conclusion or resolution that seems acceptable. While we might consider individual narratives and the narrative strategies through which they invest recovery with a normative force, what becomes even more significant are the ways repetition functions in them. To encounter one narrative’s version of a happy ending is to encounter a possibility. When seemingly every narrative shares that same vision of how a story of self-harm should end—specifically that *self-harm itself* should end—there are no longer possibilities, only the necessity of recovery. Within such endless repetition there is very little solace to be found for anyone whose own narrative does not meet such a requirement.

*Anesthesia* aligns perfectly with this so-called acceptable narrative structure. Even within a very small number of scenes, no more than four or five, Sophie’s narrative remains on an arc of recovery, as if such a structure is the only one possible. The final image of Sophie is on the street outside her apartment, with Professor Zarrow.^29^ At his prompting comment that “you made me a promise,” she reluctantly pulls her curling iron from her rucksack and hands it over to him. In some ways the scene shies away from the certainty of cessation, with Sophie noting that “I can just get another one … I can use cigarettes.” Nevertheless, she does give up her tool of self-harm, and its significance is evident in the scene’s physicality, as we see a long moment in which they both hold the curling iron, with her holding on without relinquishing it. At his admonition, “Sophie,” she sighs and releases her grip, saying “Thank you.” The comment has the tone of a commitment, of the very promise the professor initially invoked. It is almost as if the necessity of cessation is impossible to avoid; if Sophie can recover, and cease self-harming, after merely two conversations, what does that mean for those whose self-harm is ongoing?

Yet in many ways for our purposes the significance of this scene is not Sophie at all, or at least not just Sophie; the importance lies in the way that for a viewer like Lou, Sophie (and other characters like her) becomes simply one in an endless train of characters who self-harm and whose happy ending is recovery.^30^ Talking about our relation to the norm, Ahmed uses the metaphor of a path, saying “A path is made by the repetition of the event of the ground ‘being trodden’ upon…. The lines that direct us … are in this way performative: they depend on the repetition of norms and conventions, of routes and paths taken, but they are also created as an effect of this repetition.”^31^ I would argue that recovery from and cessation of self-harm is one such path, one that is so well-trodden that the story simply slips along it, as does Sophie. Through this very ease, the shame of falling outside of that path intensifies. In trying to contain Sophie’s shame, in promising its end and future absence, the narrative defers or transmits that shame. The shame doesn’t disappear through its resolution—rather it is simply left, waiting, for those of us to whom it belongs to pick it up. Those of us who are un-recovered, who have strayed from the path of recovery, or even who might stray from that path in the future.

We might take it for granted that the point of narratives around self-harm, what every person naturally desires, is to make self-harm as un-shameful as possible. Indeed, such an orientation might be found within this essay, and within the accounts of participants it contains. Yet this does not mean it is universal. Siobhan, when I asked her what a “good” representation of self-harm might be, said:

The things that stick out for me is when I’ve either been scared as a result or ashamed as a result of self-harm … I think to represent it, it’s all those, those things. It’s the walking to A&E, you know, at 3am in the morning because you can’t stop a cut bleeding. Or it’s like developing disgusting rashes because you’re not like watching your wounds or you’re deliberately like not taking care of them. It’s the kind of … It’s not as clean as it comes across. But that said as well … you know … is that contributing to kind of stigma about it then? I don’t know.

As we see from this quotation, the parts of self-harm that can feel most urgent and real are the parts that are shameful, because shame is an important emotion. For all that it is an emotion we might instinctively draw back from, that doesn’t mean we always must or should follow that instinct. When I watch Sophie, in *Anesthesia*, I want to turn away. I want to say that this is too embarrassing to watch, to look at. I want to say that this is inaccurate, that it gets it wrong, and that besides it is a bad film in ways entirely separate from its representation of self-harm. Yet I am drawn to her as well, to her awkwardness and her attempts to explain something that is hard. I am pulled into the language of her body which communicates her distress when the words of the script fail.

The many aspects of shame with which this viewing experience is invested gives the film some form of meaning to me that is hard to explain. Suffice to say that for all the film’s frustrations and failures, the ways it is hackneyed and sentimental and self-satisfied, I’m not certain that those failures are failures of too much shame. Sophie’s shame, the shame of *being* Sophie, and the shame of *watching* Sophie (this spiraling, multiple shame that so easily blends together) makes me want to flee, but it makes me want to be close to her as well. Because I would never feel that shame so intensely if it wasn’t also my own, if I wasn’t invested in these practices and these ways of feeling and being, in what it means to be a person who self-harms. If I didn’t care for that, and therefore for her, then it wouldn’t matter.

I have attempted to introduce nuance and complexity to the oft taken-for-granted relationship between shame and self-harm, and to argue that through fictional representations we can see that relationship being actively constructed. I have suggested that shame has functioned to *connect*, to create a vector between individual self-harming subjects and fictional characters, imagined others, and presumed collectives. I have noted the narrative strategies and techniques through which this occurs: through the collection of shame-full characteristics within a pre-existing self-harming “type”; through narrative structures that foreground visibility and exposure and then associate that exposure with nakedness, vulnerability, and excess; through positioning self-harming characters within and as representative of the artistic failures of specific genres; and finally through the enforcement of social and narrative norms via narrative repetition which creates “acceptable” and “unacceptable” trajectories of self-harm.

Participants in this study described shame’s presence somewhat ambivalently; while it was clearly a significant affect or emotion, their comments neither reflected a belief that self-harm itself is inherently shameful nor described a simple process whereby they saw a representation of self-harm and identified its similarity to their own life, and felt shame. Rather, they repeatedly experienced an engagement with a fictional representation in which the narrative positioned them, as self-harming subjects, in relation to self-harm’s shamefulness through specific characteristics, actions, and genres. It is through this positioning that they felt shame, a shame that suggests a judgment of themselves and their actions they didn’t necessarily accept or agree with, and yet still caused them pain. In understanding this process, and exploring some of the representational tools by which it was accomplished in *Anesthesia*, we can see more clearly how shame functions in relation to self-harm. Through this understanding we can perhaps, as Siobhan invites us, come to be close to shame: close to its multiplicity, its ambivalence, and even its excess, both in fiction and in our own lives.

## Supplementary Material

Title Page

## Notes

1 Potter, “Grounding for Understanding,” 218.

2 Lloyd, Blazely, and Phillips, “Stigma Towards Individuals”; Brown and Kimball, “Cutting to Live.”

3 Chandler, *Self-Injury, Medicine and Society*, 114.

4 Gurung, “Bodywork,” 41.

5 Ahmed, *Cultural Politics of Emotion*, 104.

6 Ahmed, *Cultural Politics of Emotion*, 105.

7 Ahmed, *Cultural Politics of Emotion*, 105.

8 Munt, *Queer Attachments*, 2.

9 Faulkner, *Ethics of Survivor Research*.

10 Mauthner and Doucet, “Analyzing Maternal and Domestic Voices”; Brown and Gilligan, “Listening for Voice.”

11 Dolezal and Lyons, “Health-related Shame,” 257.

12 One of the interview participant age groups was “18–25 years old.”

13 *Anesthesia*, 7:50 to 9:08.

14 Mitchell, *Writing Shame*, 152; Sheils and Walsh, *Shame and Modern Writing*, 5.

15 *Anesthesia*, 7:50 to 9:18.

16 Mitchell, *Writing Shame*, 67; Dolezal, *Body and Shame*, 24.

17 Leys, *From Guilt to Shame*, 128.

18 *Anesthesia*, 36:40 to 39:30.

19 Lou’s comments echo the oft-made analysis that shame requires positive investment. This formulation consciously echoes Sedgwick and Frank’s landmark theorization of shame (“Shame in the Cybernetic Fold”).

20 Bray, “Anorexic Body,” 414.

21 Melodrama has long been considered a shameful genre, its examples having what Frances Wilson describes as a “reputation … for embarrassing their audiences” and whose pleasures of sensationalism and excess have, as Marcia Landy notes, come to signify a failure of intellect, taste, and even ethics (Wilson, “Exaggerated Woman,” 198; Landy, Introduction). Feminist reappraisals of the genre have highlighted the misogynistic nature of such critiques, and have claimed melodrama as an important site of literary study. However, I would suggest that this re-evaluation has not entirely rehabilitated the genre’s reputation with the general public, and so its characterization as shameful remains relevant.

22 Gledhill, “Prologue,” ix.

23 *Anesthesia*, 42:15 to 44:50.

24 Mitchell, *Writing Shame*, 160.

25 This is not to suggest that all the texts participants mentioned carry the same aesthetic failures as *Anesthesia*, but rather to explore one way that self-harming characters might come to take on or exemplify the most derided characteristics of the melodramatic texts within which they are positioned; this would certainly be interesting to explore further in other texts.

26 Dolezal, “Shame Anxiety,” 3.

27 Kokkola, “Sparkling Vampires”; Trewavas, Hasking, and McAllister, “Representations of Non-suicidal.”

28 O’Donnell, Sapouna, and Brosnan, “Storytelling,” 4.

29 *Anesthesia*, 1:08:40 to 1:10:10.

30 This is not to claim that every representation of self-harm ends in recovery, as this would be self-evidently untrue. However, Lou is certainly correct that this is an extremely common narrative trajectory; a detailed discussion of the bifurcation of narrative futurities for self-harming subjects between death and recovery can be found in the fourth chapter of my doctoral thesis. See Heney, “Our Stories, Our Selves.”

31 Ahmed, *Queer Phenomenology*, 16.
