# Supplementary material for: Stories of Shame, Stories for Shame: Fiction and Self-harm
Source: Lit Med. Author manuscript; Available in PMC 2026 Jan 19. (PMC7618646; doi:10.1353/lm.2025.a975552)
Supplement: Title Page [file EMS211966-supplement-Title_Page.docx]

Title Page

Title: Stories of Shame, Stories for Shame: Fiction and Self-harm

Author Information:

Veronica Heney, Discovery Research Platform for Medical Humanities, Durham University

[Veronica.heney@durham.ac.uk](mailto:Veronica.heney@durham.ac.uk)

<https://orcid.org/0000-0002-5497-7656>

Abstract

That self-harm is a shameful practice is often taken for granted. However, recent sociological work has called attention to the way this shamefulness is actively constructed through narrative. This essay takes up that call, with a particular focus on fictional representations. It takes an interdisciplinary approach, weaving together a close reading of Tim Blake Nelson’s 2015 film Anesthesia with data from interviews of people with experience of self-harm. Specifically, it attends to the location of shame in a “type” of self-harming character, the significance of visibility and exposure in narratives of self-harm, the function of genre in invoking or communicating shame, and the uncertain relationship between destigmatization and the enforcement of norms. It thus explores shame as contingent, relational, and actively brought into being.
